# Comparison of energy expenditure measurements by a new basic respiratory room vs. classical ventilated hood

**DOI:** 10.1186/s12937-023-00903-3

**Published:** 2023-12-20

**Authors:** Timia Van Soom, Wiebren Tjalma, Ulrike Van Daele, Nick Gebruers, Eric van Breda

**Affiliations:** 1 Department of Rehabilitation Sciences & Physiotherapy, Faculty of Medicine and Health Sciences, Research Group MOVANT, Multi-Disciplinary Metabolic Research Unit (M2RUN), University of Antwerp, Universiteitsplein 1, Antwerp, 2610 Belgium; 2 Antwerp University Hospital (UZA), Multidisciplinary Breast Clinic, Wilrijkstraat 10, Edegem, 2650 Belgium; 3 Antwerp University Hospital (UZA), Multidisciplinary Edema Clinic, Wilrijkstraat 10, Edegem, 2650 Belgium; 4 Department of Medicine, Faculty of Medicine and Health Sciences, University of Antwerp, Universiteitsplein 1, Antwerp, 2610 Belgium; 5 OSCARE, Organization for Burns, Scar Aftercare and Research, Van Roiestraat 18, Antwerp, 2170 Belgium

**Keywords:** Indirect calorimetry, Respiratory room, Ventilated hood, Energy expenditure, Nutritional assessment

## Abstract

**Background:**

Nutritional support is often based on predicted resting energy expenditure (REE). In patients, predictions seem invalid. Indirect calorimetry is the gold standard for measuring EE. For assessments over longer periods (up to days), room calorimeters are used. Their design makes their use cumbersome, and warrants improvements to increase utility. Current study aims to compare data on momentary EE, obtained by a basic respiration room vs. classical ventilated hood. The objective is to compare results of the basic room and to determine its ^1)^reliability for measuring EE and ^2)^sensitivity for minute changes in activity.

**Methods:**

Two protocols (P1; P2)(*n* = 62; 25 men/37 women) were applied. When measured by hood, participants in both protocols were in complete rest (supine position). When assessed by room, participants in P1 were instructed to stay half-seated while performing light desk work; in P2 participants were in complete rest mimicking hood conditions. The Omnical calorimeter operated both modalities. Following data were collected/calculated: Oxygen uptake ($\dot{\mathrm{V}}$ O_2_(ml/min)), carbon dioxide production ($\dot{\mathrm{V}}$ CO_2_ml/min), 24h_EE (kcal/min), and respiratory exchange ratio (RER). Statistical analyses were done between modalities and between protocols. The agreement between 24h_EE, $\dot{\mathrm{V}}$ O_2_ and $\dot{\mathrm{V}}$ CO_2_ obtained by both modalities was investigated by linear regression. Reliability analysis on 24h_EE determined ICC.

**Results:**

No significant differences were found for 24h_EE and $\dot{\mathrm{V}}$ O_2_. $\dot{\mathrm{V}}$ CO_2_ significantly differed in P1 + P2, and P2 (hood > room). RER was significantly different (hood > room) for P1 + P2 and both protocols individually. Reliability of 24h_EE between modalities was high. Modality-specific results were not different between protocols.

**Discussion/conclusion:**

The room is valid for assessing momentary EE. Minute changes in activity lead to a non-significant increase in EE and significant increase in RER. The significant difference in $\dot{\mathrm{V}}$ CO_2_ for hood might be related to perceived comfort. More research is necessary on determinants of RER, type (intensity) of activity, and restlessness. The design of the room facilitates metabolic measurements in research, with promising results for future clinical use.

## Introduction

In 1955, the nutritional needs of patients were already recognized [1] and remain a topic of interest today. For example, recently malnutrition was frequently observed in hospitalized patients with COVID-19 [2]. The urge to strive for adequate nutritional support in clinical situations continues to be of major concern [3]. Malnutrition remains underrecognized and undertreated in many patients [4–6]. Total energy expenditure (TEE) determines a person’s individual energy requirement, which is ideally met by total energy intake (TEI). When TEE and TEI match, an energy balance is reached. A positive (TEI > TEE) or negative (TEI < TEE) balance induces weight gain or -loss, respectively [5]. It has been reported that the presence of a disease and/or its treatment disturbs the energy balance by affecting the body’s energy expenditure (EE) (e.g. chemotoxicity during cancer treatment). If nutritional needs are not met, the resulting disbalance causes alterations in body weight (BW), seen as mass changes of the different body components (body composition (BC)). Deteriorating changes in fat mass (FM) and fat free mass (FFM), specifically skeletal muscle mass, will negatively affect general health, Quality of Life and activities of daily living [6].

Various methods have been developed to determine EE. A common tool is the predictive Harris-Benedict Equation (HB_Eq_), which is based on anthropometric variables and easy applicable in clinical practice [7]. Predictive equations are valid in the overall healthy population [8], however, EE is often underestimated in hospitalized patients due to their condition/nature of the disease [4, 9]. Hence, accurate analysis of EE is of utmost importance to ward energy balance. Indirect calorimetry (IC) is the gold standard because of its high sensitivity and precision [10]. Newer technology and better understanding of how to interpret measurements should lead to more frequent use, consequentially improving individualized nutritional support in clinical settings as well.

To date, different indirect calorimeters are commercially available and can, depending on the setup, be used for cardiopulmonary exercise tests or the analysis of (momentary/resting) EE. For measuring EE, either a ventilated hood or respiratory room is used. A disadvantage of the hood for bedside applications is the short period (30 min) of possible measuring time due to increased build-up of arousal. Additionally, it is often more accurate and informative to measure EE under free-living conditions (e.g., during sleep), requiring prolonged periods of measuring time. Whole-room calorimeters have been designed for the latter, in which EE measurements up to several days are feasible [11, 12].

Although room calorimeters are highly accurate and referred to as gold standard, they are expensive because of their technological design, and require specialized staff. In addition, the fact that they are often located away from a patients’ whereabouts makes their use in hospital settings cumbersome. Recently, a basic version of a full respiration room has been developed (*Maastricht Instruments*, Maastricht, The Netherlands). This room is easy to operate by standard indirect calorimetry devices and allows bed rested patients to be measured during their hospital stay. A major advantage of this system over the use of a ventilated hood is that patients can be followed throughout the day or night resulting in more reliable measures of EE.

Therefore, the major aims of the present study were 1) to compare results of the momentary measure of the rate at which a person uses energy, further referred to as momentary energy expenditure, assessed by the basic version of a respiratory room compared to gold standard assessments with ventilated hood, and 2) to determine the room’s sensitivity for minute changes in activity under semi free-living conditions.

## Materials and methods

This cross-sectional comparison study was conducted at the Multidisciplinary Metabolic Research Unit (M^2^RUN) of the research group Movant (University of Antwerp (BE); department of Rehabilitation Sciences and Physiotherapy). Comparison between EE measured by room and ventilated hood is done by means of two protocols (P1 and P2). In P1, minimal activity (light desk work) is performed inside the room to assess its sensitivity for minute changes in EE related to activity. In P2, EE is measured inside the room under identical resting conditions, which allows to compare its results with hood modality. The study is approved by the medical ethical committee of the University of Antwerp/Antwerp University Hospital (B300201942189).

### Subjects

Sixty-two subjects were recruited through social media or direct contact with the researchers and enrolled after providing a written informed consent. Between January and December 2019, 32 subjects participated in P1, between January and February 2022, 30 subjects in P2. Inclusion criteria were: adults (> 18 years), healthy (absence of (chronic) disease), no recent (< 1 month) medical treatment (e.g. surgery) or current use of pharmacological substances. Subjects were excluded when one of these criteria was not met. Demographic characteristics can be found in Table 1.

**Table 1:** Demographic characteristics of all participants, and comparison of subjects between protocols

Characteristics of study participants				
Parameter (*n* = m/w)	**Protocol 1 + 2** (mean ± SD) **62 (25/37)**	**Protocol 1** (mean ± SD) **32 (10/22)**	**Protocol 2** (mean ± SD) **30 (15/15)**	***p*-value**
**Age (y)**	33.2 ± 11.0	32.7 ± 11.1	33.8 ± 10.9	0.692
**Height (cm)**	174.3 ± 8.6	174.8 ± 8.4	173.9 ± 8.9	0.690
BW (kg)	71.8 ± 13.4	72.1 ± 15.1	71.4 ± 11.6	0.841

*SD* Standard deviation, *n* Number of subjects, *m* men, *w* women, *y* year, *BH (cm)* Body height in centimeter, *BW (kg)* Body weight in kilogram;

^*^Significant difference

Significance α < 0.05

### Procedures

For P1 and P2, two IC measurements 1) respiration room and 2) ventilated hood were consecutively performed (1.5 h in total). After arrival at the research facility, a period of relative rest (± 15 min) was set before beginning of the measurements so that the participants could acclimate. Relative rest is defined as a state of being, where tasks can be performed, albeit without physical effort (which has repercussions on the respiratory rate). Assessments were done under semi-fasting conditions and executed on the same day. Subjects were asked to refrain from heavy exercise at least 12-h before and solid meals or snacks 3-h prior the study. Water was allowed ad libitum. The order of measurements was decided by simple randomization. Flipping a coin assigned the subject to the first measuring device (head = room vs. tail = hood) after which the second device was automatically designated. After each respiratory room assessment, a 15 min washing-out time was installed to refresh the air inside, and to limit measurement errors related to build-up of respiratory gases. In addition, we predicted EE based on the HB_Eq_ for all participants in P1 and P2 to collate with hood measurements [8], so as to compare the results obtained by the room with valid hood measurements. Following HB_Eq_ was used:

$$E{E}_{H{B}_{eq}\left(men\right)}=66.4730+\left(13.7516*BWkg\right)+\left(5.0033*BHcm\right)-(6.7550*age)$$

$$E{E}_{H{B}_{eq}\left(women\right)}=655.0955+\left(9.5634*BWkg\right)+\left(1.8496*BHcm\right)-(4.6756*age)$$

### Indirect calorimetry

All IC measurements were done with the same an open circuit diluted flow calorimeter (*Omnical IV*, *Maastricht Instruments*, Maastricht, The Netherlands), with the only difference in measurements was the size of the “headspace” per modality (hood: 0,03m^3^; room: 14m^3^) (average temperature (C°): 22.5 ± 3.1 C°; average humidity (%): 46.6 ± 3.3%; light: 500 lm (ambient light)). The Omnical is the fourth generation calorimeter and developed based on methods for whole‐room calorimetry [13–16]. Calibration of the device was performed automatically every 30 min with span gas (18% O_2_ and 0.8% CO_2_) and nitrogen gas (100%). Validation of the system by methanol combustion was performed weekly as described previously [15]: The theoretical value for the respiratory exchange ratio (RER;$\dot{\mathrm{V}}$ CO_2_/$\dot{\mathrm{V}}$ O_2_) was 0.667, based on the ratio of produced CO_2_ to utilized O_2_ from the burning of methanol from the equation: 2CH_3_OH + 3O_2_ → 2CO_2_. In brief, the methanol burner has been set to burn 0.2 g/min, equivalent to the production of 150mlCO_2_/min and a consumption of 225mlO_2_/min. Since an error percentage is dependent on burn-rate, the expression of the error limit in absolute ml/min is preferred, hence the limit values of 7.5 and 11 ml/min for CO_2_ and O_2_ respectively (5% of 150 ml CO_2_ and 5% of 225 ml O_2_). For validation in the range of EE, methanol (pro‐analysis, 99.8%; *Merck Millipore BV*, Amsterdam, The Netherlands) was burned at a target $\dot{\mathrm{V}}$ O_2_ rate of 225 ml/min [15]. All measurements were normalized to standard temperature and pressure dry (STPD) values by measuring temperature, humidity, and pressure. Data of the methanol burning tests can be found in (Appendix Table 7).

#### Ventilated Hood

**Fig. 1:**
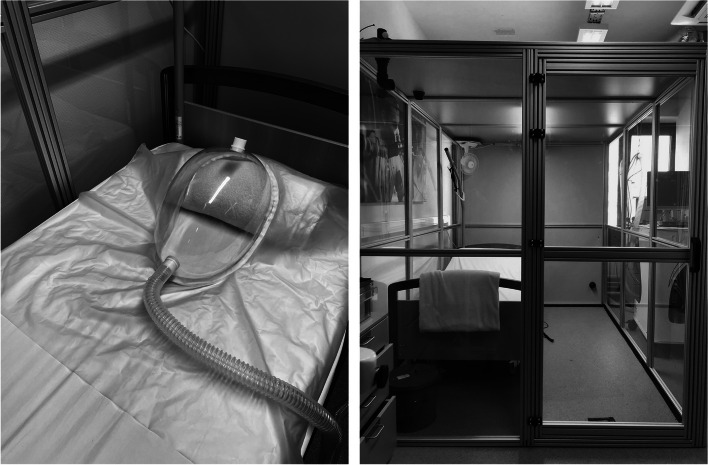
Modalities used for measuring energy expenditure. Left: Ventilated hood; Right: Basic respiratory room

Hood measurements were performed inside the room, with open door to prevent built up of environmental CO_2_ concentrations. This way, subjects could remain in the same resting facilities and environmental conditions. Momentary EE was assessed after 3 h of fasting as approximation of resting EE (REE) [17]. Assessment of momentary EE by ventilated hood was identical for P1 and P2 (Fig. 1). All subjects were asked to lay still on a bed in supine position with the hood (0,03m^3^) placed over their heads. The hood served as reservoir collecting $\dot{\mathrm{V}}$ O_2_ and $\dot{\mathrm{V}}$ CO_2_. A continuous flow of fresh air is directed through the hood and all in- and outgoing air is analyzed for O_2_ and CO_2_ concentrations, while the airflow through the system is measured, determining gas concentrations for in- and expired air with a representative resolution of ≤ 0.001% [15]. The flow rate was equal between P1 and P2 (Table 2). Inspired air (environment) samples were taken every 2 min, while expired air was analyzed every other 2 min as well as continuously. The measurement with ventilated hood lasted 30 min and data were provided every 5 s. Only data of the last 20 min were used to ascertain a balanced recording of gas exchange rates ($\dot{\mathrm{V}}$ O_2_ and $\dot{\mathrm{V}}$ CO_2_) as fluctuations are often seen in the beginning of the measurement due to a slow decrease towards a resting level. From sitting or standing to resting in supine position, there is a decrease in EE which typically takes 10 min to achieve a steady state [17, 18]. The Weir equation (Weir, 1949) was used to convert $\dot{\mathrm{V}}$ O_2_ and $\dot{\mathrm{V}}$ CO_2_ to EE values. The RER, indicative for substrate utilization, was calculated as an average over the whole selected measurement to limit errors due to intra-individual variations. Data were collected by the Omnical device to the 6^th^ decimal as kcal/min (EE_Hood_;kcal/min) and recalculated (1^st^ decimal) by the researchers into kcal in 24 h (24h_EE_Hood_;kcal). Results obtained by ventilated hood were used as gold standard [8].

**Table 2:** Flow rate in room and hood

**Flow rate**			
**Subjects of P1 or P2**	**Protocol 1** (mean ± SD)	**Protocol 2** (mean ± SD)	***p*-value**
**All subjects of P1** (*n* = 32) **or P2** (*n* = 30)			
Flow rate_Room	177.9 ± 21.0	179.3 ± 2.3	0.706
Flow rate_Hood	91.4 ± 12.5	89.9 ± 10.9	0.610

*Legend*. *SD* Standard deviation, *n* Number of subjects, *P1* Protocol 1, *P2* Protocol 2; flow rate in liter/minute

Significance = α < 0.05

#### Respiratory room

A basic version of a respiration room (14m^3^) was built by using plexiglass windows in an aluminum frame (Fig. 1). The windows were made airtight with rubber seals. A ventilator was placed on the ceiling to mix the air in the room. An air inlet was placed through the ceiling while the air outlet was placed at the bottom of one of the walls. Room temperature and humidity were measured with the Rotronic probe HC2A-S (*Rotronic*, Basserdorf, Germany). Validation was identical as described above for the hood. An extra methanol validation was performed inside the room by burning methanol for 60 min in a fireproof bucket. The flow rate was equal between P1 and P2 (Table 2).

The room served as reservoir collecting $\dot{\mathrm{V}}$ O_2_ and $\dot{\mathrm{V}}$ CO_2_. Because of the larger volume of the room, the measurement lasted 60 min and data were provided every minute. Only results of the last 50 min were used. $\dot{\mathrm{V}}$ O_2_ and $\dot{\mathrm{V}}$ CO_2_ were converted by the Omnical to EE values (Weir, 1949). Data on momentary EE were collected to the 6^th^ decimal as kcal/min (EE_Room_;kcal/min) and recalculated by the researchers (1^st^ decimal) to kcal in 24 h (24h_EE_Room_;kcal). The RER was calculated as an average over the whole selected measurement, hence limiting errors related to intra-individual variations.

The respiratory room measurement of momentary EE was different between P1 and P2. For P1, subjects were instructed to stay in a seated position on a bed while performing light desk work on a laptop computer (minimal activity). For P2, subjects were asked to lay still imitating the subject’s position when measured by hood.

### Statistical analysis

Quantitative variables are expressed as mean ± SD. All data collected during system calibration and in P1 and P2 were checked for normality with the Kolmogorov–Smirnov test, normality plot and boxplot. Validation of the system is reported by accuracy and variability (measure of precision); Accuracy is reported as mean % difference of expected vs. observed gas exchange rates ($\dot{\mathrm{V}}$ O_2_, $\dot{\mathrm{V}}$ CO_2_). Variability is reported as the standard deviation (SD) of the expected vs. observed % difference [19]. A variability of < 3% was selected as upper limit treshold of reliability, as previously described [20].

The independent samples T-test was used to compare subject characteristics between participants of P1 and P2 to avoid bias related to the study population. The paired-samples T-test compared the 24h_EE measured under the hood with EE_HB_Eq_ for all subjects together (Protocol 1 + 2) and for subjects of P1 and P2 individually (Table 8. in Appendix). To analyze comparability between modalities, a paired samples T-test between room and hood modality was performed on all subjects together (Protocol 1 + 2) for 24h_EE, $\dot{\mathrm{V}}$ O_2_, $\dot{\mathrm{V}}$ CO_2_, and RER, as well as for the subjects participating in the individual protocols (P1 or P2). Next, the independent samples T-test examined the variability of 24h_EE, $\dot{\mathrm{V}}$ O_2_, $\dot{\mathrm{V}}$ CO_2_, and RER between P1 and P2 for hood and room, to confirm the equal resting state of the first modality and analyze the ability of the room to detect minimal activity.

Proportional bias and equality of the range of results between room and hood for the participants of P2 was investigated by linear regression analysis for 24h_EE, $\dot{\mathrm{V}}$ O_2_, and $\dot{\mathrm{V}}$ CO_2_, with a Bland–Altman plot to analyze the agreement between modalities under similar resting conditions. A reliability analysis on 24h_EE between room and hood for all subjects (Protocol 1 + 2) and study samples of P1 and P2 separately was conducted to analyze the intraclass correlation coefficient (ICC; two-way mixed – absolute agreement). All statistical tests were executed two-sided (Significance: α = 0.05). Statistical analyses were performed with the use of SPSS software (SPSS v28, IBM Business Analytics, New York, USA).

## Results

### General characteristics of the subjects

Sixty-two (62) subjects, 32 in P1 (10men(m)/22women(w)) and 30 in P2 (15 m/15w) were recruited. The demographic characteristics and comparison between the participants of P1 and P2 combined (Protocol 1 + 2), and P1 and P2 independently are shown in Table 1. No significant differences were found between the subjects of both protocols.

### Validation of Omnical system

The mean percentage difference between expected and observed results for analyzing $\dot{\mathrm{V}}$ O_2_ was 0.22% with 0.25% variability rate. For $\dot{\mathrm{V}}$ CO_2_, the mean difference was -1.67% with 1.87% variability, while this was -1.86% and 1.81% respectively for RER (Table 3). Variability rate between the assessment of gas concentrations and RER by Omnical and expected values was < 3%.

**Table 3:** Validation of Omnical

**Results system validation by methanol combustion**			
**Parameter**	**Accuracy** Mean	**Precision** SD	**95% CI**
**%diff ** $\dot{\mathbf{V}}$** O**_**2**_	0.22	0.25	[-0.2566—0.7717]
**%diff ** $\dot{\mathbf{V}}$** CO**_**2**_	-1.67	1.87	[-4.6493—1.9417]
**%diffRER**	-1.86	1.81	[-4.4039—1.4786]

*Legend*: %diff: Percentage difference between expected vs. observed values

$\dot{V}$ O_2_ Rate of oxygen uptake (ml/min), $\dot{V}$*CO*_*2*_ Rate of carbon dioxide production (ml/min), *RER* Respiratory exchange rate, *SD* Standard Deviation, *95%CI* 95% confidence interval

### Comparison of momentary energy expenditure, calorimetric gas measurements and respiratory exchange rate

#### Room vs. Hood

##### Momentary expenditure

24h_EE was not different between room and ventilated hood conditions for all subjects together (room vs. hood P1 + P2: *p* = 0.543) and for all participants in P1 (*p* = 0.158) and P2 (*p* = 0.281) independently (Table 4).

**Table 4:** Comparison of momentary energy expenditure based on indirect calorimetric gas analysis between room and ventilated hood

Energy expenditure, oxygen uptake & carbon dioxide production & respiratory exchange ratio ROOM vs. HOOD			
**Subjects of P1 and P2 room vs. hood**	**Room** (mean ± SD)	**Hood** (mean ± SD)	***p*-value**
**Subjects of P1 + P2** (*n* = 62)			
24h_EE	1597.2 ± 262.3	1585.2 ± 260.0	0.543
$\dot{\mathrm{V}}$ O_2_	230.3 ± 37.7	226.0 ± 36.4	0.139
$\dot{\mathrm{V}}$ CO_2_	181.5 ± 32.0	187.9 ± 35.5	0.031*
RER	0.79 ± 0.05	0.83 ± 0.05	< 0.001*
**Subjects of P1 room vs. hood** Minimal activity vs. rest	**Room** (mean ± SD)	**Hood** (mean ± SD)	***p*-value**
**Subjects of P1** (*n* = 32)			
24h_EE	1619.8 ± 271.6	1574.5 ± 283.6	0.158
$\dot{\mathrm{V}}$ O_2_	234.0 ± 39.1	224.3 ± 39.4	0.051
$\dot{\mathrm{V}}$ CO_2_	182.6 ± 33.4	186.1 ± 39.7	0.391
RER	0.78 ± 0.05	0.83 ± 0.06	< 0.001*
**Subjects of P2 room vs. hood** Rest vs. rest	**Room** (mean ± SD)	**Hood** (mean ± SD)	***p*-value**
**Subjects of P2** (*n* = 30)			
24h_EE	1573.0 ± 254.4	1596.5 ± 236.7	0.281
$\dot{\mathrm{V}}$ O_2_	226.4 ± 36.4	227.8 ± 33.6	0.614
$\dot{\mathrm{V}}$ CO_2_	180.4 ± 30.9	189.9 ± 31.0	0.029*
RER	0.80 ± 0.04	0.83 ± 0.05	0.015*

*Legend*: *SD* Standard deviation, *P1* Protocol 1, *P2* Protocol 2, *n* Number of subjects, *24h_EE* Energy expenditure in 24 h (kcal), $\dot{V}$*O*_*2*_ Rate of oxygen uptake (ml/min), $\dot{V}$*CO*_*2*_ Rate of carbon dioxide production (ml/min), *RER* respiratory exchange ratio

^*^Significant difference

Significance α < 0.05

##### Gas analysis ($\dot{\mathbf{V}}$ O_2_ and $\dot{\mathbf{V}}$ CO_2_) and respiratory exchange rate

$\dot{V}$ O_2_ did not differ significantly between room and hood for all subjects together (*p* = 0.139), and for P1 (*p* = 0.051) and P2 (*p* = 0.614) separately. $\dot{V}$ CO_2_ was significantly different between room and hood in P1 + P2 (*p* = 0.031) and in P2 independently (*p* = 0.029). No significant difference was found for $\dot{\mathrm{V}}$ CO_2_ in P1 (*p* = 0.391). The RER of all subjects together (P1 + P2) was significantly higher for the hood measurement compared to the room (*p* < 0.001). The same was seen in P1 (*p* < 0.001) and P2 (*p* = 0.015) independently **(**Table 4).

### Detection of minimal activity

#### Protocol 1 vs. Protocol 2

##### Momentary energy expenditure

No significant differences were found for 24h_EE between both protocols (P1 vs. P2) used under hood (*p* = 0.742) or room (*p* = 0.487) conditions (Table 5).

**Table 5:** Modality-specific comparison of momentary energy expenditure based on indirect calorimetric gas analysis between protocol 1 and protocol 2

**Energy expenditure, oxygen uptake, carbon dioxide production & respiratory exchange ratio** **HOOD P1 vs. HOOD P2**			
**Subjects of P1 vs. P2** Rest vs. rest	**P1** (mean ± SD)	**P2** (mean ± SD)	***p*-value**
**P1** (*n* = 32) **vs. P2** (*n* = 30)			
Hood 24h_EE	1574.5 ± 283.6	1596.5 ± 236.7	0.742
Hood $\dot{\mathrm{V}}$ O_2_	224.3 ± 39.4	227.8 ± 33.6	0.707
Hood $\dot{\mathrm{V}}$ CO_2_	186.1 ± 39.7	189.9 ± 31.0	0.679
Hood RER	0.83 ± 0.06	0.83 ± 0.05	0.970
**Energy expenditure, oxygen uptake, carbon dioxide production & respiratory exchange ratio** **ROOM P1 vs ROOM P2**			
**Subjects of P1 vs. P2** Minimal activity vs. rest	**P1** (mean ± SD)	**P2** (mean ± SD)	***p*-value**
**P1** (*n* = 32) **vs. P2** (*n* = 30)			
Room 24h_EE	1619.8 ± 271.6	1573.0 ± 254.4	0.487
Room $\dot{\mathrm{V}}$ O_2_	234.0 ± 39.1	226.4 ± 36.4	0.436
Room $\dot{\mathrm{V}}$ CO_2_	182.6 ± 33.4	180.4 ± 30.9	0.796
Room RER	0.78 ± 0.05	0.80 ± 0.04	0.122

*Legend*: *SD* Standard deviation, *P1* Protocol 1, *P2* Protocol 2, *n* Number of subjects, *24h_EE* Momentary energy expenditure in 24 h (kcal), $\dot{V}$*O*_*2*_ Rate of oxygen uptake (ml/min), $\dot{V}$*CO*_*2*_ Rate of carbon dioxide production (ml/min), *RER* Respiratory exchange ratio

^*^Significant difference. Significance α < 0.05

##### Gas analysis ($\dot{\mathbf{V}}$ O_2_ and $\dot{\mathbf{V}}$ CO_2_) and respiratory exchange rate

Values of V̇O_2_ and V̇CO_2_ were also not significantly different between protocols (P1 vs. P2) for both modalities (Hood: V̇O_2_: *p* = 0.707, V̇CO_2_: *p* = 0.679; Room: V̇O_2_: *p* = 0.43, V̇CO_2_: *p* = 0.796).The RER for all subjects, either measured by hood or room, did not show a significant difference between P1 and P2 (Room: *p* = 0.122; hood: *p* = 0.970) (Table 5).

### Linear regression analysis and Bland–Altman plot for P2

**Fig. 2:**
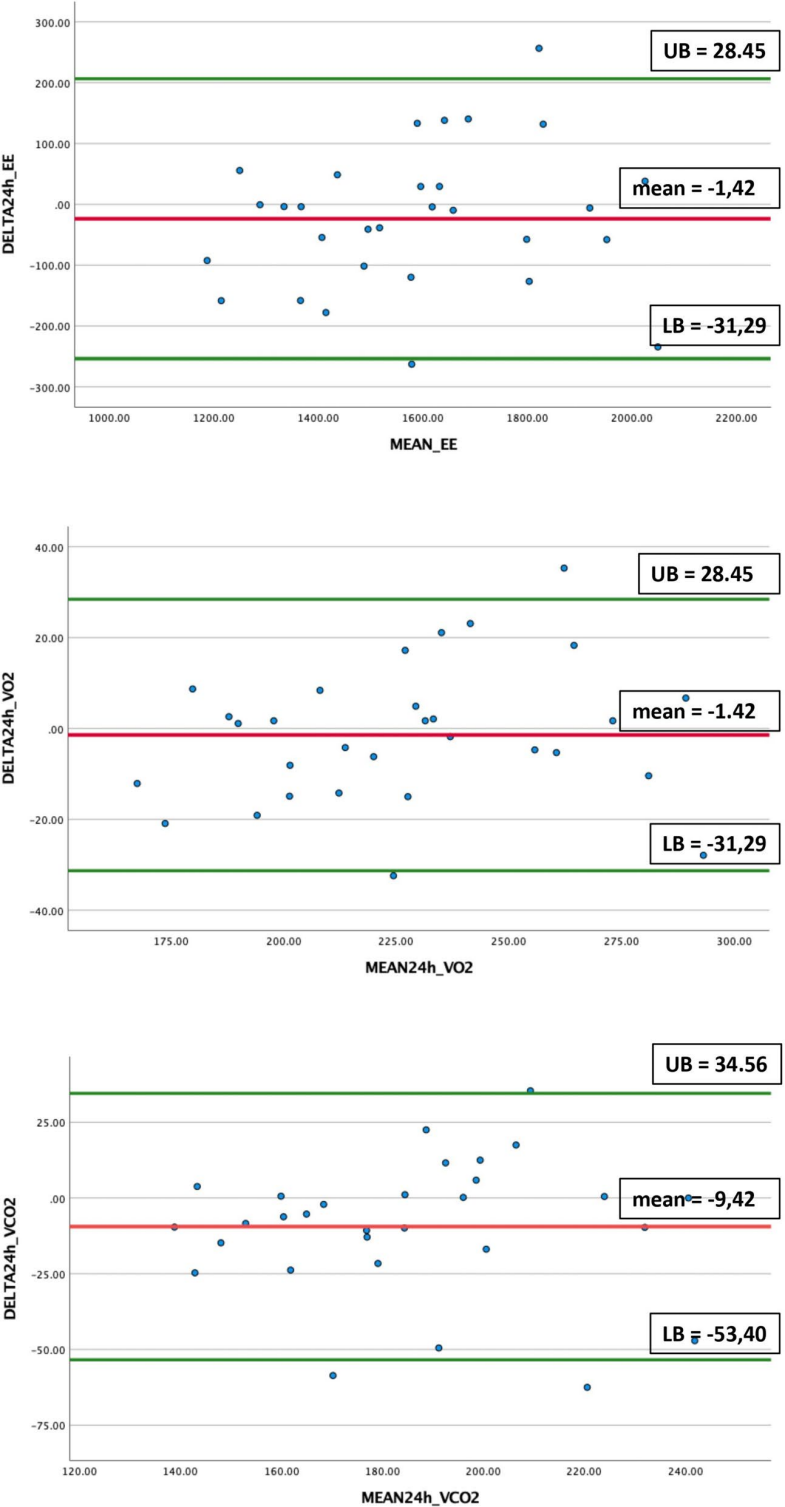
Bland–Altman plot of the agreement between modalities. Upper: Bland–Altman plot for 24h_EE measured by room and hood; Middle: Bland–Altman plot for VO_2_ measured by room and hood; Lower: Bland–Altman plot for VCO_2_ measured by room and hood; DELTA24h_EE: Difference between 24h_EE measured by hood and room for subjects of P1 + P2; MEAN24h_EE: Mean of 24h_EE measured by hood and room for subjects of P1 + P2; DELTA_ VO_2_: Difference between VO_2_ measured by hood and room for subjects of P1 + P2; MEAN_ VO_2_: Mean of VO_2_ measured by hood and room for subjects of P1 + P2; DELTA_ VCO_2_: Difference between VCO_2_ measured by hood and room for subjects of P1 + P2; MEAN_ VCO_2_: Mean of VCO_2_ measured by hood and room for subjects of P1 + P2; UB: Upper boundary; LB: Lower boundary

No proportional bias was found for 24h_EE, $\dot{\mathrm{V}}$ O_2_ and $\dot{\mathrm{V}}$ CO_2_ for all subjects in P2. Regression analysis of the mean difference between 24h_EE in room and hood (delta 24h_EE) on the average of the two methods (mean 24h_EE) showed a non-significant unstandardized coefficient of 0.076 (*p* = 0.412). For the mean $\dot{\mathrm{V}}$ O_2_, the unstandardized coefficient was 0.086 (*p* = 0.310), and for mean $\dot{\mathrm{V}}$ CO_2_ -0.001 (*p* = 0.994) (Table 6). Figure 2 shows the Bland–Altman plots of the mean differences and averages of 24h_EE, $\dot{\mathrm{V}}$ O_2_ and $\dot{\mathrm{V}}$ CO_2_, respectively, obtained by P2.

**Table 6:** Detection of proportional bias between results of P2

**Linear regression**		
**Subjects of P2 (*n* = 30)**	**Unstandardized coefficient (B)**	***p*-value**
** MEAN24h_EE**	0.076	0.412
** MEAN_VO**_**2**_	0.086	0.310
** MEAN_VCO**_**2**_	-0.001	0.994
**Intraclass correlation coefficient**		
**24h_EE ROOM VS. HOOD**	**Average measure (ICC)**	**95% CI**
** All subjects** (*n* = 62)	.906	[0.844-0.943]
** Protocol 1** (*n* = 32)	.883	[0.762-0.943]
** Protocol 2** (*n* = 30)	.939	[0.873-0.971]

*Legend*: *n* Number of subjects, *MEAN24h_EE* Mean energy expenditure over 24 h for Room and Hood, *MEAN_VO*_*2*_ Mean rate of oxygen for Room and Hood, *MEAN_VCO*_*2*_ Mean rate of carbon dioxide for Room and Hood, *MEAN_RER* Mean respiratory exchange ratio for room and hood, *Room* Respiratory room modality, *Hood* Ventilated hood modality, *ICC* Intraclass correlation coefficient

^*^Significant difference

Significance α < 0.05

### Reliability between room and hood for momentary energy expenditure and gas concentrations

A high degree of reliability was found for 24h_EE in room and hood in all subjects (P1 + P2). The average ICC for was 0.906 with a 95% confidence interval (CI) of 0.844 – 0.943. Both protocols (P1 and P2) also showed a high degree of reliability with an ICC of 0.883 (CI: 0.762 – 0.943) in P1, and 0.939 (CI: 0.873 – 0.971) in P2 (Table 6).

## Discussion/conclusion

Respiratory rooms are the current gold standard for analyzing EE in free living conditions. To determine if the basic respiratory room is valid for assessing EE, we compared the measurement of EE (kcal/min) recalculated to 24h_EE (kcal), based on indirect calorimetric gas exchange parameters ($\dot{\mathrm{V}}$ O_2_**,** and $\dot{\mathrm{V}}$ CO_2_), between the new room and ventilated hood in healthy adults. For this purpose, we used a high-end indirect calorimeter that has been validated as measuring device [15, 20]. Secondly, we investigated if the room was sensitive to detect small changes in free-living conditions by means of two protocols (P1 and P2). In P1 the subjects were seated in an upright position in a hospital bed and were allowed to perform light deskwork on a laptop computer, whereas in P2 subjects were asked to lay still in supine position mimicking the resting condition as measured under the ventilated hood.

### Comparison between the new basic respiratory room and ventilated hood

Analysis of momentary energy expenditure by calorimetric gas measurements.

#### Comparison between room and hood

The present study found no significant differences in 24h_EE between the respiratory room and ventilated hood measurements. A good ICC related to the measurement of EE supports this finding (all subjects, and participants of P1 and P2 separately). The same applies to V̇O_2_ and V̇CO_2_, where no significant differences between both measuring devices were discovered.

Yet in general, a higher but non-significant V̇O_2_ was noticed in the room compared to the hood while the V̇CO_2_ was higher as measured under hood conditions. The fact that the room has a larger volume might explain the higher V̇O_2_ generally seen in the room. For the hood, hyperventilation most likely causes an increase in V̇CO_2_, a finding which is not new. As the hood is in the form of a small, confined space, accepting the canopy could bring difficulties as agitation might build-up. One should realize that the aim of measured EE is to reflect the caloric need as closely as possible. Hence assessments obtained from shorter measurements (as is the case for the hood) therefore always lead to an EE that is extrapolated and not fully accounts for the true variation during one circadian cycle [21]. The conditions under the hood can possibly result in higher values for V̇CO_2_, as seen in our study, and might consequentially lead to a misinterpretation of EE, resulting in non-adequate nutritional strategies (especially in clinical patients). From our study, we can conclude that the basic respiratory room is a valid device for assessing EE and can therefore be used for continuous measurements over longer time intervals, consequentially ameliorating nutritional support.

#### Detection of minimal activity

No difference present in 24h_EE between hood assessments of P1 and P2, and for the room assessments of P1 and P2. This motivates the assumption that the subject’s conditions for hood and room were both comparable between protocols although minimal activity was performed inside the room during P1. It can be suggested that light deskwork has similar metabolic demands (reflected by EE) compared to supine position which is in accordance with previous results of Miles-Chan et al. [22] who reported that, under ventilated hood conditions, no difference between the supine and seated position were observed. Another reason could be that the room is not sensitive enough to detect a change in minimal activity. However, since 24h_EE room in P1 was higher (although not significantly) compared to P2, it might be that the type of activity was not rigorous enough to lead to a significant increase in EE, still, a minimal change was detected by the room.

Alongside the metabolic demand of the system comes substrate (nutrient) oxidation, indicated by RER, to maintain energy homeostasis. A change in the body’s physiological state (e.g. active (light desk work) vs. rest) is therefore expected to be accompanied by a change in RER [23]. While EE between modalities was similar, RER between hood and room did differ significantly (hood > room in P1 + P2, P1, and P2 separately). Looking at room and hood separately, a small non-significant difference in room-RER was seen between protocols (P1 < P2), while the hood displayed similar results on RER (P1 = P2).

The RER is a proxy measure for the oxidative capacity of muscles to get energy. When $\dot{\mathrm{V}}$ O_2_ (due to activity) or $\dot{\mathrm{V}}$ CO_2_ (due to agitation/hyperventilation) increase, respectively a lower and higher RER is seen [24]. Comparing room-RER with hood-RER, it can be suggested that agitation/hyperventilation underneath the hood with consequently a higher $\dot{\mathrm{V}}$ CO_2_ partially explains the significant difference in RER between measurement devices. Additionally, we further hypothesize that active movements bring changes in gas concentrations forth, which can be detected by the room, affecting RER. Comparing room-$\dot{\mathrm{V}}$ O_2_ of P1 and P2, higher concentrations were found in P1 (minimal activity). Consequently, the room’s RER in P1 was smaller (although not significant) compared to P2. Since the allowance of light desk work inside the respiratory room varied between both protocols, we suggest that the active movements accompanying light deskwork might have been a decisive factor for the difference between the room-RER in both protocols, as subjects were allowed to move freely with their upper torso and arms. In addition, other subject-related variabilities can contribute to differences as well, such as hormonal status (menstrual status), previous level of exercise, or food intake prior to the 3 h of fasting. Further investigation on other specific determinants is warranted [24].

Although of clinical value, respiratory rooms are sparsely available due to high costs in infrastructure and the need of experienced and trained staff, therefore mostly used in academic research settings. The technological design of the basic respiratory room improves mobility and allows for measurements of EE under semi free-living conditions on a specific ward instead of transferring patients towards a stationary calorimeter. Accurate insights in the energy requirements can eventually lead to more individualized, patient-centered nutritional support.

### Limitations

The findings of this study should be considered in the light of some limitations. First, we did not take BC, more specific muscle mass (cfr. FFM) into account. This might have led to possible errors related to interpretation of the results as muscle mass is a large determinant of EE. FFM should therefore always be assessed concordantly by e.g. BodPod. More, the small sample size in both P1 and P2 may have affected the results, studies on larger groups are necessary to confirm our findings. Also, we did not correct for food intake. As the minimal fasting time was set at 3 h, some participants were measured early in the morning after > 8 h of fasting. An inventory of food intake by a self-reported nutritional diary or standardized diet prior to the measurement should account for possible bias related to individual food intake. Next, we did not use any wearable movement sensors or heart rate monitor to detect minimal activity such as the postulated upper body movements. In accordance, subject’s preference or perceived comfort levels were not analyzed, as well as respiration rate to assess e.g. hyperventilation. Finally, inter-individual varieties that could have introduced aberrant results were not considered. Future research with preferably a controlled nutritional intake, general health inventory, and physiological aspects of energy metabolism (e.g. imaging data and/or markers in muscle and brown adipose tissue, menstrual phase), is needed to optimize the use of the respiratory room.

## Conclusion

We conclude that the basic version of the respiratory room proofs to be valid to measure EE in research, and opens doors for future use in clinical practice. A minute change in activity resulted in an increase in EE, although not significant. Substrate utilization (RER) did differ significantly when the activity changed. Results of this study can be used to facilitate the implementation of IC in nutritional research, and lead to future use for developing individual nutritional programs.

## Data Availability

The data supporting the findings of this study are available upon reasonable request from the corresponding author. Data are not shared openly to protect privacy of study participants. Data are located in a controlled access data storage at the University of Antwerp.

## Appendix

**Table 7:** Results of methanol combustion

No. Methanol test	o $\dot{\mathbf{V}}$ O_2_	e $\dot{\mathbf{V}}$ O_2_	%$\dot{\mathbf{V}}$ O_2_	o $\dot{\mathbf{V}}$ CO_2_	e $\dot{\mathbf{V}}$ CO_2_	%$\dot{\mathbf{V}}$ CO_2_	oRER	eRER	%RER
1	268.2	268.1	0.0	178.9	176.7	1.2	0.7	0.7	1.2
2	255.6	254.5	0.3	238.9	244.7	-2.4	0.7	0.7	-2.7
3	228.1	227.9	0.1	153.1	157.4	-2.7	0.7	0.7	-2.8
4	302.2	301.9	0.1	211.5	208.5	1.4	0.7	0.7	1.3
5	291.5	290.5	0.4	195.3	200.7	-2.7	0.7	0.7	-3.0
6	249.0	249.2	-0.18	166.0	172.1	-3.6	0.7	0.7	-3.5
7	244.2	244.1	0.0	230.5	237.6	-4.0	0.7	0.7	-3.0
8	266.2	265.5	0.3	175.5	180.6	-3.0	0.7	0.7	-3.1
9	292.6	291.3	0.5	196.0	201.0	-2.5	0.7	0.7	-2.9
10	234.0	233.9	0.1	158.0	159.0	-0.7	0.7	0.7	-0.8
11	220.3	219.3	0.5	149.9	147.0	1.9	0.7	0.7	1.5
12	223.9	222.2	0.8	150.3	153.5	-2.1	0.7	0.7	-2.9
13	272.1	272.8	-0.3	182.0	190.9	-4.7	0.7	0.7	-4.4
14	229.2	228.0	0.5	154.8	157.4	-1.8	0.7	0.7	-2.2
15	296.6	296.0	0.2	200.0	201.3	-0.6	0.7	0.7	-0.8
16	233.3	233.2	0.1	156.0	161.0	-3.1	0.7	0.7	-3.2
17	247.7	246.8	0.4	169.6	170.4	-0.4	0.7	0.7	-0.8

*Legend*: *o* observed measure, *e* expected measure, *%* Percentage difference between expected vs. observed values, $\dot{V}$*O*_*2*_ Rate of oxygen uptake (ml/min), $\dot{V}$*CO*_*2*_ Rate of carbon dioxide production (ml/min), *RER* Respiratory exchange rate

**Table 8:** Comparison between measured (hood) and predicted EE (HBeq)

24h_EE hood vs. EE_HB_Eq_			
**Subjects of P1 and P2**	**Hood** (mean ± SD)	**HB**_**Eq**_ (mean ± SD)	***p*****-value**
**P1 + P2** (*n* = 62)			
24h_EE	1585.2 ± 260.0	1609.5 ± 250.2	0.243
**Subjects of P1**	**Hood** (mean ± SD)	**HB**_**Eq**_ (mean ± SD)	***p*****-value**
**P1** (*n* = 32)			
24h_EE	1574.5 ± 283.6	1598.5 ± 261.4	0.469
**Subjects of P2**	**Hood** (mean ± SD)	**HB**_**Eq**_ (mean ± SD)	***p*****-value**
**P2** (*n* = 30)			
24h_EE	1596.5 ± 236.7	1621.1 ± 241.6	0.333

*Legend*: *24h_EE* Energy expenditure recalculated to 24 h, *EE_HB*_*Eq*_ Energy expenditure predicted by Harris-Benedict equation, *P1* Protocol 1, *P2* Protocol 2, *P1* + *P2* Protocol 1 + 2, *n* Number of subjects

Significance α < 0.05

## Abbreviations

REE: Resting energy expenditure
BC: Body composition
BW: Body weight
CO_2_: Carbon dioxide
EE: Energy expenditure
FFM: Fat free mass
FM: Fat mass
HB_Eq_: Harris-Benedict equation
IC: Indirect calorimetry
ICC: Intraclass correlation coefficient
O_2_: Oxygen
P_1_: Protocol 1
P_2_: Protocol 2
(24h_)EE: Energy expenditure (in 24 h)
RER: Respiratory exchange ratio
TEE: Total energy expenditure
TEI: Total energy intake
